# Application of a Novel Strategy of Engineering Conditional Alleles to a Single Exon Gene, *Sox2*

**DOI:** 10.1371/journal.pone.0045768

**Published:** 2012-09-27

**Authors:** Nikolaos Mandalos, Marannia Saridaki, Jessica Lea Harper, Anastasia Kotsoni, Peter Yang, Aris N. Economides, Eumorphia Remboutsika

**Affiliations:** 1 Stem Cell Biology Laboratory, Institute of Molecular Biology and Genetics, Biomedical Sciences Research Centre “Alexander Fleming”, Vari, Greece; 2 Regeneron Pharmaceuticals Inc., Tarrytown, New York, United States of America; Universitätsklinikum Carl Gustav Carus an der Technischen Universität Dresden, Germany

## Abstract

**Background:**

The Conditional by Inversion (COIN) method for engineering conditional alleles relies on an invertible optimized gene trap-like element, the COIN module, for imparting conditionality. The COIN module contains an optimized 3′ splice site-polyadenylation signal pair, but is inserted antisense to the target gene and therefore does not alter transcription, until it is inverted by Cre recombinase. In order to make COIN applicable to all protein-coding genes, the COIN module has been engineered within an artificial intron, enabling insertion into an exon.

**Methodology/Principal Findings:**

Therefore, theoretically, the COIN method should be applicable to single exon genes, and to test this idea we engineered a COIN allele of *Sox2*. This single exon gene presents additional design challenges, in that its proximal promoter and coding region are entirely contained within a CpG island, and are also spanned by an overlapping transcript, *Sox2Ot*, which contains *mmu-miR1897*. Here, we show that despite disruption of the CpG island by the COIN module intron, the COIN allele of *Sox2* (*Sox2^COIN^*) is phenotypically wild type, and also does not interfere with expression of *Sox2Ot* and *miR1897*. Furthermore, the inverted COIN allele of *Sox2*, *Sox2^INV^* is functionally null, as homozygotes recapitulate the phenotype of *Sox2^ßgeo/ßgeo^* mice, a well-characterized *Sox2* null. Lastly, the benefit of the eGFP marker embedded in the COIN allele is demonstrated as it mirrors the expression pattern of *Sox2*.

**Conclusions/Significance:**

Our results demonstrate the applicability of the COIN technology as a method of choice for targeting single exon genes.

## Introduction

Intronless (single exon) genes are thought to be evolutionary innovations, whose formation via reverse transcription–mediated mechanisms represents an important route of evolution for tissue-specific functions in animal cells [1], [2]. Approximately 12% of the human and 13.4% of the mouse protein-coding genes are intronless [3]–[6], and include genes that encode for regulatory proteins and components of signal transduction pathways [7], histones [8]–[11], G Protein-coupled Receptors [3] and transcription factors such as the *Sox* (SRY-related HMG box) family [12]–[14].

*Sox2* is a well-characterized and important example of a single exon gene. Sox2 pairs with tissue-specific partners [15] to impart and maintain pluripotency [16] and multipotency [14], [17] during development and homeostasis [18]. *Sox2* null embryos fail to form the epiblast and die at E5.5 [19]. However, even reduction in *Sox2* levels to 25–30% relative to the wild type leads to pathological phenotypes in mice. These include neurodegeneration in the cortical region and hippocampus [20], hypoplasia of optic nerves and chiasmata and variable microphthalmia [21], failure of nasal placode induction [22], failure of taste buds to mature [23], malformation of the epithelium lining the conducting airways in the lung [24], enlargement of the lateral ventricles at E14.5 [25], and immature differentiation of cochlea hair follicles [26].

From a gene structure standpoint, *Sox2* presents a complex locus rich in genetic elements, including an overlapping transcript [27], a putative microRNA [28], and a CpG island [29]–[31] [32]. The combination of a well-conserved compact locus with overlapping transcripts and regulatory elements [33]–[39], together with the apparent need to maintain proper levels of Sox2 for organogenesis and homeostasis, underscore the difficulties associated with designing conditional alleles for *Sox2*. We hypothesized that a recently developed method for generating conditional alleles – Conditional by Inversion (COIN) – might present a better choice over simple floxing of *Sox2*, and generated the corresponding conditional-null allele, *Sox2^COIN^*. We show that this method is successful in that the *Sox2^COIN^* allele starts as wild type, and it is converted into a null by the action of Cre, at which point, the expression of *Sox2* is replaced by that of a marker, *eGFP*. This work indicates that the COIN method can be applied to single exon genes and provide a new design modality that can be adopted for other genes like *Sox2*.

## Results

### Generation of the *Sox2^COIN^* Allele

**Figure 1:**
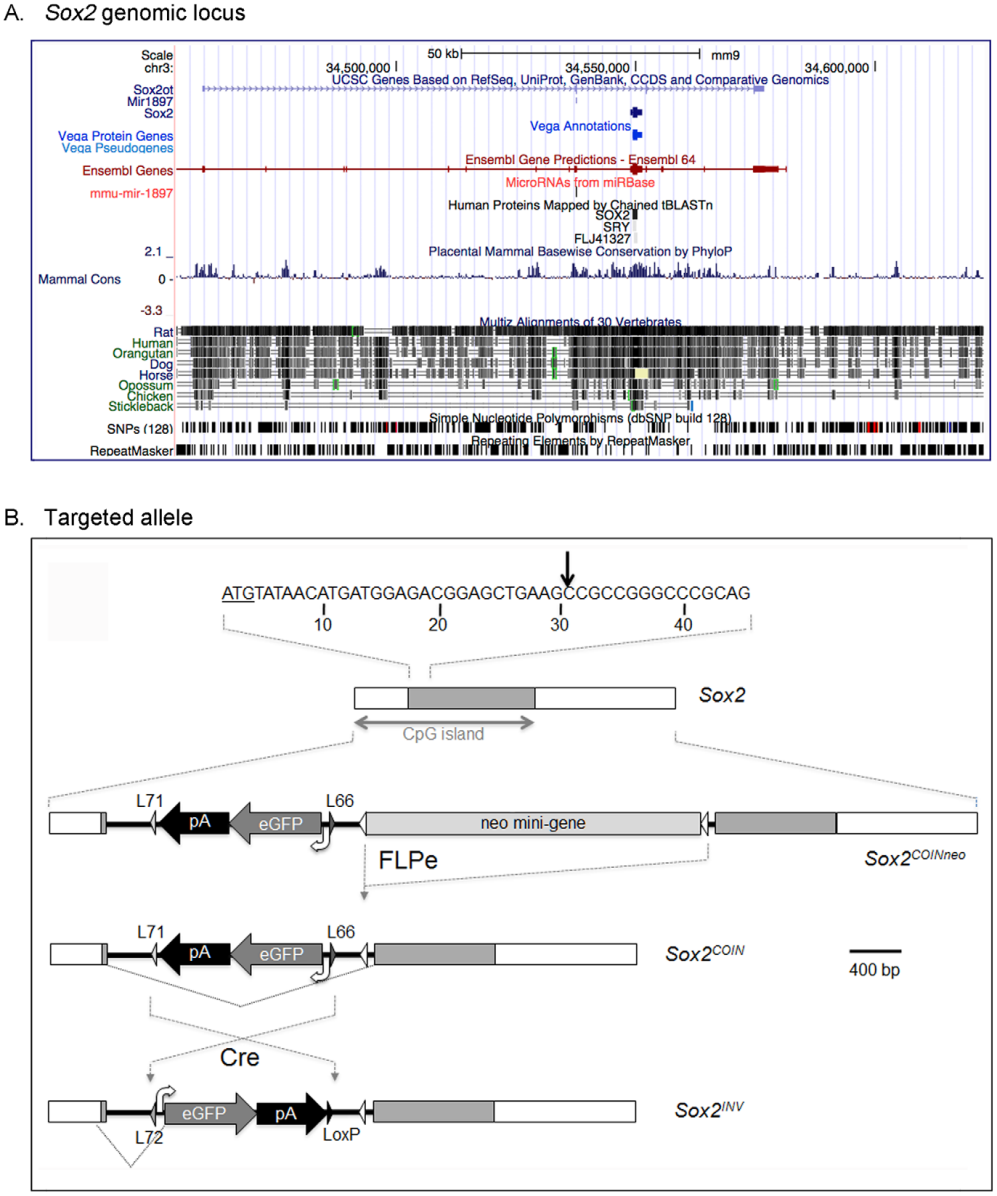
Targeting strategy generating a COIN allele of *Sox2*. (**A**) Schematic representation of the mouse *Sox2* locus indicating the relative location of the exon on chromosome 3, as well as that of *mir1897*, the non-coding RNA *Sox2ot*, and CpG islands in the genomic region. The degree of conservation of the locus sequence between mammalian species (ECRs) is indicated. Adapted from http://genome.ucsc.edu. (**B**) Schematic representation of the *Sox2^COIN^* allele. The COIN module intron is inserted after the 30^th^ nucleotide of *Sox2*’s coding region (i.e. coordinate 34549367 on Chromosome 3) splitting the single exon of Sox2 into two exons and also dividing the CpG island. The COIN module is comprised of an optimized gene trap-like element composed of the 3′ splice region of the rabbit beta globin gene (HBB_RABIT), followed by *eGFP* (lacking an initiating ATG) and the polyadenylation region from HBB_RABIT, all placed in the antisense strand. The COIN module has been flanked with *Lox71* and *Lox66* sites in a mirror image orientation, thereby enabling inversion by Cre. For BHR and targeting, a FRT-flanked *neo* cassette has been incorporated into the COIN intron. After targeting, *neo* is removed to give rise to the *Sox2^COIN^* allele. The COIN module is antisense to *Sox2*, and hence it predicted not to interfere with expression of *Sox2*. However, after inversion of the COIN module to the sense strand, transcription terminates around the polyadenylation region of the COIN module, and as a result expression of *Sox2* is replaced by *eGFP*.

*Sox2* (ENSMUSG00000074637) is a single exon gene encoding a 319 amino acid protein. The *Sox2* locus contains several features that render it complex from the standpoint of engineering modified alleles (**Fig. 1A**). To begin with, *Sox2’s* proximal promoter and coding region comprise a CpG island [15]. Furthermore, the *Sox2* exon is contained with the intron of a long non-coding RNA (ncRNA), termed *Sox2* overlapping transcript (*Sox2ot*) or “non-protein coding RNA 43”, which also contains mmu-miR1897 (miR1897) [40]. *Sox2ot* is transcribed from the same strand as *Sox2* but its molecular and biological functions remains elusive. *Sox2ot* transcript is expressed in mouse embryonic stem cells and in other tissues, including the nervous system, where *Sox2* is also highly expressed [41], while an isoform of *Sox2ot*, *Sox2dot*, located around 500 base pairs upstream of *Sox2*, was detected exclusively in adult mouse brain [27]. Because of this complexity, *Sox2* is a challenging locus to apply conditional mutagenesis, and therefore presents a stringent test for new methods of allele design, such as COIN.

The COIN method relies on an optimized gene trap-like element, referred to as the COIN module [42]. The COIN module is comprised of a *3′ splice region-reporter cDNA-polyadenylation region* optimized to function as an efficient transcriptional block, and it is flanked by *Lox71* and *Lox66* sites are in a mirror image configuration to enable Cre-mediated inversion [43]. In order to generate conditional-null alleles, the COIN module is placed in a position antisense to the target gene, either within a native intron, or an exon. The latter is made possible by embedding the COIN module within an artificial intron – the COIN module intron – and using that intron to split the target exon into two operational halves [42].

To generate *Sox2^COIN^*, the COIN module intron was inserted directly into the single exon of *Sox2* (Figure 1B), after the 30^th^ nucleotide of *Sox2*’s open reading frame, splitting the single *Sox2* exon into two exons. The COIN module lies inertly within the antisense strand of *Sox2*, stealth to transcription. Upon Cre-mediated inversion into the sense orientation, the *Sox2^COIN^* allele is converted into a null allele, *Sox2^INV^*. This is accomplished by the COIN module abrogating transcription of full length Sox2, effectively replacing it with expression of the COIN module’s eGFP reporter. The expression of *eGFP* in place of *Sox2* is controlled by *Sox2*’s promoter and regulatory elements, and enables visual identification of the inversion event at the tissue and cellular level. The functionality of the allele was assessed *in vivo* in a series of experiments that assessed whether *Sox2^COIN^* is a truly wild type allele, and whether *Sox2^INV/INV^* recapitulate the null phenotype, while providing a useful marker that faithfully reproduces the expression profile of *Sox2*.

### *Sox2^COIN^* is Wild Type in Homozygosis

**Figure 2:**
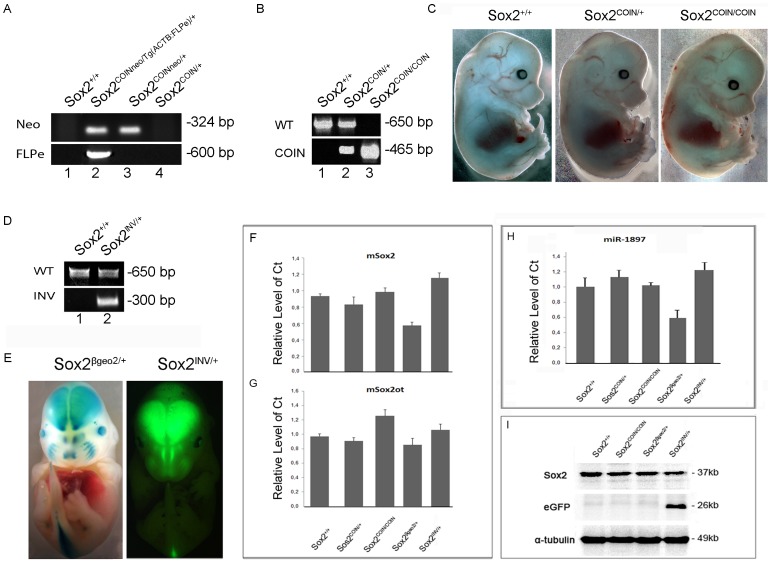
*Sox2^COIN^* is a functional conditional allele. (**A**) Efficient removal of the *neo* cassette by Flpe recombinase to generate a *Sox2^COIN/+^* allele. *Sox2^+/+^* (lane 1), *Sox2^COIN/+^* (lane 2), *Sox2^COIN/COIN^* (lane 3), *Sox2^INV/+^* (lane 4), *Sox2^βgeo2/+^* (lane 5) E14.5 mice. PCR genotyping of (1) *Sox2^+/+^*, (2) *Sox2^COIN/+^ Tg(ACTB:FLPe)*, (3) *Sox2^COINneo/+^*, (4) *Sox2^COIN/+^*. (**B**) PCR genotyping of *Sox2^+/+^* (lane 1), *Sox2^COIN/+^* (lane 2), *Sox2^COIN/COIN^* (lane 3) (genomic DNA from tail biopsies). (**C**) E14.5 *Sox2^COIN/COIN^* embryos are morphologically indistinguishable from *Sox2^+/+^* and *Sox2^COIN/+^*. (**D**) Efficient inversion of the COIN module to generate *Sox2^INV/+^* mice. PCR-based genotyping of (1) *Sox2^+/+^* and (2) *Sox2^INV/+^* (genomic DNA from tail biopsies). (**F, G**) *Sox2* and *Sox2ot* qPCR analysis in *Sox2^+/+^*, *Sox2^COIN/+^*, *Sox2^COIN/COIN^*, *Sox2^βgeo2/+^* and *Sox2^INV/+^* E14.5 embryos. Cyclophilin was used as a control label. Data are presented as the mean+SEM (n = 3–6) for each genotype. The COIN module does not affect expression of *Sox2* prior to inversion. Additionallly, the COIN module does not appear to affect the expression level of *Sox2ot* either in *Sox2^COIN/COIN^* or in *Sox2^INV/+^* embryos. (**H**) *MiR1897* qPCR analysis in *Sox2^+/+^*, *Sox2^COIN/+^*, *Sox2^COIN/COIN^*, *Sox2^βgeo2/+^* and *Sox2^INV/+^* E14.5 embryos. (**I**) Sox2 protein analysis. Western blot showing Sox2 and eGFP protein detected with specific antibodies in whole protein extracts from *Sox2^+/+^*, *Sox2^COIN/+^*, *Sox2^COIN/COIN^*, *Sox2^βgeo2/+^*, and *Sox2^INV/+^* E14.5 embryos. The COIN module does not affect Sox2 protein levels prior to inversion.

**Figure 3:**
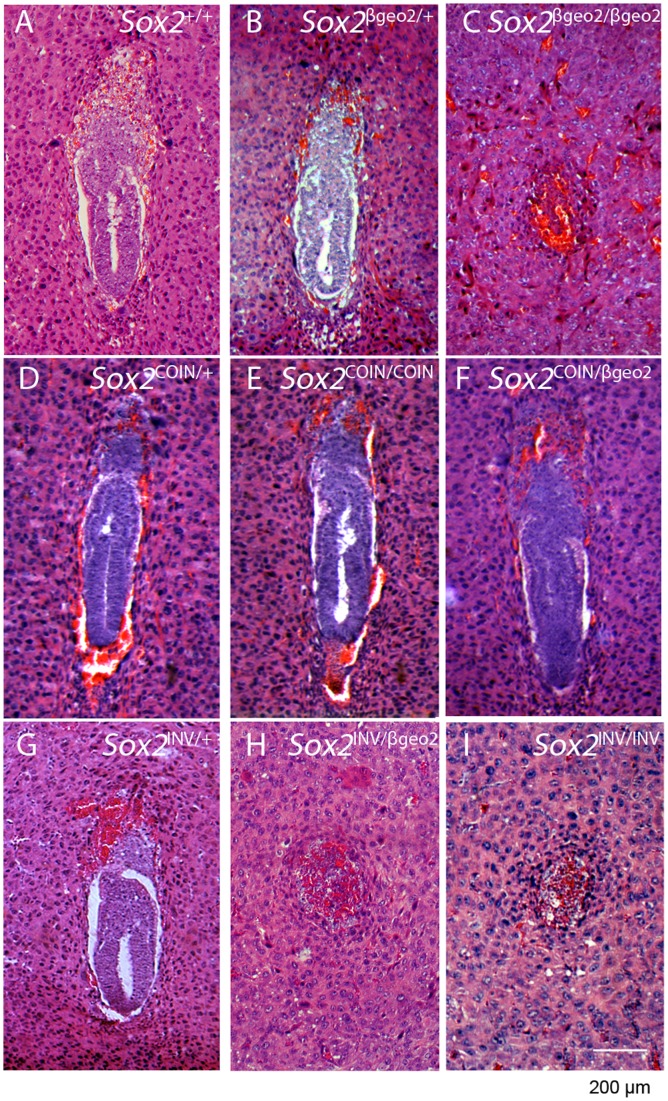
*Sox2^INV^* is a null allele. Histological analysis of 6.5-dpc embryos from *Sox2^βgeo2/+^*, *Sox2^COIN/+^*, *Sox2^INV/+^* intercrosses. *Sox2^+/+^* (**A**), *Sox2^βgeo2/+^* (**B**), *Sox2^COIN/+^* (**D**), *Sox2^COIN/COIN^* (**E**), *Sox2^COIN/βgeo2^* (**F**), and *Sox2^INV/+^* (**G**) are all phenotypically normal. In contrast, *Sox2^INV/INV^* (**I**) and *Sox2^INV/βgeo2^* (**H**) embryos form disorganized extraembryonic tissues, but fail to form the epiblast, effectively phenocopying *Sox2^ βgeo2/βgeo2^* (**C**), and displaying the phenotype previously described for *Sox2* homozygous-null embryos. Sections were stained with hematoxylin and eosin (H & E).

Offspring of *Sox2* ^COIN/+^ intercrosses were born in Mendelian ratios and no lethality was observed in embryos, newborn pups and adults (Table 1). Homozygote mice fed normally, showed no abnormal behavior and they had normal weight in adulthood (data not shown). Macroscopic analysis of E14.5 *Sox2^+/+^*, *Sox2^COIN/+^*, and *Sox2^COIN/COIN^* mice showed that the COIN module does not affect normal embryonic mouse development (Figure 2C). These phenotypic observations are further corroborated by the result that *Sox2^COIN/βgeo2^* E6.5 embryos were morphologically indistinguishable from *Sox2* ^+/+^ or *Sox2* ^βgeo2/+^ embryos derived from a *Sox2^βgeo2/+^* with *Sox2^COIN/COIN^* cross (Figure 3B, F), where *Sox2^βgeo2^* is a null allele of *Sox2* [19] (see below). Furthermore, examination of *Sox2* mRNA (Figure 2F) and Sox2 protein (Figure 2H) expression levels show no apparent difference between the three genotypic classes, *Sox2^+/+^*, *Sox2^COIN/+^*, and *Sox2^COIN/COIN^*, demonstrating that the COIN module has no effect on the expression of *Sox2*. Thus, by all of these criteria – heritability, phenotype, expression of mRNA and protein –*Sox2^COIN^* behaves as a wild type allele.

**Table 1:** Analysis of progeny from *Sox2* ^COIN/+^ intercrosses#.

Genotypic distribution in live progeny*					
	Live	Dead	*Sox2^+/+^*	*Sox2^COIN/+^*	*Sox2^COIN/COIN^*
Age	No	No	No (%)	No (%)	No (%)
P21	95	0	27 (25%)	40 (50%)	28 (25%)

Genotyping of *Sox2* ^COIN/+^ heterozygous intercross progeny.

# Data collected from mice in C57BL6 background.

* Genotypes were assessed by PCR of genomic tail DNA.

### The Expression of *Sox2ot and miR1897* are Unaffected in *Sox2^COIN/COIN^* Mice

To assess whether the COIN module affects *Sox2ot* RNA expression, we isolated RNA from E14.5 mouse embryos from different intercrosses and quantified *Sox2ot* RNA levels by Taqman Real-Time PCR analysis (Figure 2G). No significant difference was detected in the expression of *Sox2ot* in *Sox2^+/+^*, *Sox2^COIN/+^*, and *Sox2^COIN/COIN^*, demonstrating that the COIN module has no effect on the expression of *Sox2ot*, at least prior to inversion. Identical observations where made for *miR1897*, which is embedded in *Sox2ot* (Figure 2H).

### *Sox2^COIN^* is Efficiently Inverted by Cre to Generate *Sox2^INV^*

To assess whether we could trigger COIN inversion upon Cre expression, *Sox2^COIN/+^* adult mice were intercrossed with *Sox2*Tg(Sox2:CRE) transgenic mice to generate *Sox2^INV/+^* embryos and adult mice (Figure 2D, E). In contrast to the partial infertility phenotype that has been observed with *Sox2^ßgeo2/+^* mice [19], *Sox2^INV/+^* adult mice exhibited no obvious phenotypes and transmitted the inverted allele in Mendelian ratios (Table 2), irrespective of whether the *Sox2^INV^* allele is transmitted via the male or female germline (data not shown). More importantly, E14.5 *Sox2^INV/+^* embryos (Figure 2E) displayed vivid eGFP expression in the cerebral cortex, retina, olfactory bulb, hair follicles, olfactory epithelium and spinal cord (Figure 2E), mirroring what has been observed with X-gal stained E14.5 *Sox2^βgeo2/+^*embryos [44]. In addition, the presence of eGFP protein can be detected by Western blotting in protein extracts derived from *Sox2^INV/+^* embryos, and appears to be accompanied by a reduction in the levels of Sox2 protein, similar to what has been observed in the *Sox2^ßgeo2/+^* embryos (Figure 2I).

**Table 2:** Analysis of progeny from *Sox2^INV/+^* × *Sox2^+/+^* intercrosses#.

Genotypic distribution in live progeny*				
	Live	Dead	*Sox* 2^+/+^	*Sox2^INV/+^*
Age	No (%)	No (%)	No (%)	No (%)
E6.5	17	0	9 (53%)	8 (47%)
P21	73	0	40 (55%)	33 (45%)

Genotyping of *Sox2^INV/+^* heterozygous intercross progeny.

# Data collected from mice in C57BL6 background.

* Genotypes were assessed by PCR either from tail biopsies or whole embryos.

### *Sox2^INV^* is a Null Allele of *Sox2*

Mice carrying a loss of function mutation in the *Sox2* locus have been generated by the insertion of a βgeo cassette into the *Sox2* locus (*Sox2* ^βgeo^ [19], and *Sox2^βgeo2^* [44]). Upon homozygosis, both alleles yield *Sox*2-null embryos that fail to form the epiblast and die around implantation. To test whether *Sox2^INV/INV^* phenocopy *Sox2^βgeo2/βgeo2^*, we performed *Sox2* ^INV/+^ intercrosses (Table 3). No *Sox2* ^INV/INV^ offsprings were born. More specifically, *Sox2^INV/INV^* mutants failed to survive shortly after implantation (Figure 3I), phenocopying *Sox2^βgeo/^* ^βgeo^ and *Sox2^βgeo2/βgeo2^* embryos. Only *Sox2* ^+/+^ and *Sox2^INV/+^* embryos reach the embryonic stage of E6.5 (Figure 3A, G). Histological examination of whole decidual swellings harvested at 6.5 dpc revealed that 25% of deciduas carried abnormal implants, which had no egg cylinder structure and lacked the epithelial cells typical of epiblast (Figure 3I). Instead, many trophoblast giant cells could be identified (Figure 3C, H, I). The same phenotype is observed in *Sox2^INV/βgeo2^* embryos (Figure 3H). These results demonstrate the failure of *Sox2^INV/INV^* embryos to develop an epiblast similarly to the *Sox2^βgeo/βgeo^* and *Sox2^βgeo2/βgeo2^* mutants. Thus, the inverted COIN cassette generates a true *Sox2* null phenotype.

**Table 3:** Analysis of progeny from *Sox2^INV/+^* × *Sox2^INV/+^* intercrosses#.

Genotypic distribution in live progeny*					
	Live	Dead	*Sox2^INV/+^*	*Sox2* ^INV/INV^	*Sox2* ^+/+^
Age	No	No (%)	No (%)	No (%)	No (%)
E6.5	18	6 (33%)	5 (28%)	0	7 (29%)
P21	43	0 (0%)	21 (49%)	0	22 (51%)

Genotyping of *Sox2^INV/+^* heterozygous intercross progeny.

# Data collected from mice in C57BL6 background.

* Genotypes were assessed by PCR either from tail biopsies or embryo tissue.

## Discussion

We show here the application of COIN technology to generate a conditional-null allele for a single exon gene, *Sox2*. The COIN method was invented at least in part to overcome the challenges and limitations of traditional site-specific recombinase-based strategies such as Cre/Lox for designing conditional alleles [45]. These include the placement of Lox sites as well as the distance between them [46], defining critical exons (i.e. the exons of the gene that need to be deleted by Cre in order to bring about the desired allelic state) [47], [48], and the lack of unified strategy for including a reporter that can mark those cells that harbor the post-recombinase allele. To date, COIN has been successfully applied in generating conditional alleles for more than twenty-five protein-coding genes [42], but its applicability to single exon genes has not been tested.

Single exon genes present a design challenge for engineering conditional alleles by traditional, e.g. simple floxing, methods. First, the Lox sites should be placed in a position that does not affect the expression of the target locus, a design decision that can be complicated by the lack of specific knowledge of the exact position of promoters and regulatory elements. An additional design challenge is presented if a reporter that marks the conversion from ‘wild-type’ to null is desired. The COIN method addresses both of these challenges irrespective of gene structure by avoiding the placement of Lox and FRT sites, reporters, and other functional elements within regions upstream of the target gene’s coding sequence [42]. Instead, COIN employs an ‘exon-splitting’ artificial intron to place an optimized module – the COIN module – within a coding exon, yet in the antisense strand. Perusal of the regulatory elements mapping within single coding exon of human Sox2 suggests that the COIN intron was inserted at a position that does not result in disruption of any such elements (data not shown) As a result, the COIN module is stealth to transcription, and does not alter the expression of the modified gene. Although it is possible that introduction of the COIN module intron alters the kinetics of transcription [49]–[51], we have not examined this possibility at the single cell level; at the population level and at steady state, the level of *Sox2* mRNA as expressed from the *Sox2^COIN^* locus does not appear different to that of wild type. Lastly, because in COINs the Lox and FRT sites are placed within the artificial intron, they do not disrupt of promoters or regulatory sequences, and are also not incorporated into mRNA.

The particular choice of *Sox2* to test the COIN method’s applicability to single exon genes presented additional challenges in that the majority of *Sox2*’s single exon is contained within a CpG island, and there is also an overlapping non-coding transcript *Sox2ot*. Due to design constraints – specifically the need to place the COIN module as near the initiating ATG as possible – the COIN module intron was inserted into *Sox2*’s exon in manner that disrupts the CpG island. However, this had no apparent effect on Sox2 expression and had not apparent phenotypic consequences in the mouse. *Sox2ot* levels also remained unaltered, indicating that at least in the antisense position the presence of the COIN module has no effect on the expression of *Sox2ot*. This was evident by the normal phenotype of *Sox2^COIN/βgeo2^* embryos, in which only the COIN allele can generate wild type mRNA. This genotype should sensitize the embryo to any reduction in Sox2 levels, and thus provides a stringent comparison between the wild type allele and the COIN allele prior to inversion.

Equally important is the fact that post-inversion of the COIN module, the resulting allele, *Sox2^INV^* phenocopies the previously generated null alleles upon homozygosis. In addition, the reporter embedded in the COIN module, is expressed in a manner representative of Sox2 expression, thereby generating a tool to visualize Sox2 expression and to follow the conversion of the COIN allele into a null by Cre.

In addition to the conditional-null allele presented here, four other conditional-null alleles of *Sox2* have been published [21], [52]–[54]. All four rely on floxing of the single exon of *Sox2*, though the placement of the LoxP sites and selection cassettes (and their retention) varies among alleles. One of the main differences between these alleles and *Sox2^COIN^*, is that they do not incorporate a reporter that is activated after Cre acts on the allele. There is however a paucity of published data such as expression analysis of *Sox2*, *Sox2ot*, and *miR1897* to allow further comparisons between *Sox2^COIN^* and the previously described conditional-null alleles. Given the increasing evidence for roles that Sox2 plays in a wide range of pathological and patho-physiological conditions, assays for the normal regulation of *Sox2* expression need to be conducted in a variety of cell types. Overall, our results highlight the importance of the Conditional by Inversion technology as a method of choice in targeting intronless (single exon) genes, especially when complexity of the locus and desire for inclusion of a reporter are taken into consideration.

## Methods

### Gene Targeting

Targeted *Sox2^COINneo/+^* ES cells were generated using VelociGene™ methodology, essentially as described [55]. Briefly, the BAC-based targeting vector was assembled on bacterial artificial chromosome (BAC) RP23_406a6 that encompasses the single protein-coding exon of *Sox2* flanked by approximately 95 and 71 kb upstream and downstream respectively. The COIN module intron was introduced by bacterial homologous recombination [56] after the 30^th^ nucleotide of *Sox2*’s coding region (i.e. coordinate 34549367 on Chromosome 3, as annotated on Ensembl release 67), splitting the single coding exon of *Sox2* into two exons of 441 bp and 2016 bp respectively (Figure 1B).

### Experimental Animals

*Sox2^COINneo/+^* mice were bred with *Tg(ACTB:FLPe)* mice (Flp-deleter mice) to excise the Neo cassette and generate *Sox2^COIN/+^ Tg(ACTB:FLPe)* mice. These were bred with C57BL6 mice to bring the *Sox2^COIN^* allele into the germline. *Sox2^COIN/+^* mice were in turn bred with Tg(*Sox2*:CRE) mice to generate *Sox2^COIN-INV/+^* mice. All animals were handled in strict accordance with good animal practice as defined by the Animals Act 160/03.05.1991 applicable in Greece, revised according to the 86/609/EEC/24.11.1986 EU directive regarding the proper care and use of laboratory animals and in accordance to the Hellenic License for Animal Experimentation at the BSRC” Alexander Fleming” (Prot. No. 767/28.02.07) issued after protocol approval by the Animal Research Committee of the BSRC “Alexander Fleming” (Prot. No. 2762/03.08.05).

### Embryo Processing, Tissue Preparation and Histological Analysis

For staging of the embryos, midday of the vaginal plug was considered as embryonic day 0.5 (E0.5). E6.5 decidua and E14.5 embryos were collected and dissected in cold PBS. Tissues were fixed with 10% formalin for 24 hours at room temperature and then washed several times with 1% PBS, then placed in embedding cassettes. Paraffin sections (10 µm) were stained with Hematoxylin and Eosin (H&E) using standard procedures and mounted with xylene based mounting medium. E14.5 *Sox2* ^βgeo2/+^ LacZ staining was performed following standard protocol [19].

### Genotyping

Tail, yolk sack or embryonic tissues were isolated and processed according to previously described methodology. Deletion of the EM7p-neo-polyA FRT-flanked (FRTed) in the germline dual-purpose antibiotic/drug selection cassette by FLP recombinase was documented by genotyping PCR from adult mice tail. Neo gene was amplified with *Neo* Frw (5′-CTGAATGAACTGCAGGACGA-3′), *Neo* Rvs (5′-ATACTTTCTCGGCAGGAGCA-3′) (172 bp); FLPe with *FLP* Frw (5′-GAGAAGAACGGCATAGTGCG-3′), *FLP* Rvs (5′-GACAAGCGTTAGTAGGCACAT-3′) (600 bp). Genomic DNA from E14.5 embryos was isolated from yolk sack. In E6.5 embryos genomic DNA was isolated by scraping carefully under the stereoscope off glass slides after staining with H&E and mounting. Detachment of cover slides was done by embedding mounted slides back in xylene. Detection of *Sox2* ^COIN/+^ mice that carry the assay for the COIN cassette was performed using the following set of primers: 5′ end primer combination (338 bp), INVdiaF2 (F2): 5′ CACTTTCTACTCTGTTGAC 3′, INVdiaR2 (R2): 5′ CCTTACATGTTTTACTAG 3′, 3′ end primer combination (470 bp), INVdia(eGFP).F3 (F3): 5′CTGAAGCACTGCACGCCGTAG 3′, INVdia.R4 (R4): 5′ CTCAGAGTATTTTATCCTCATCTC 3′ (Figure 2a). PCR amplification of COIN cassette in mice and embryos E6.5 and E14.5 was also performed with the following primers: Sox2ATG407 Frw: 5′ATGTATAACATGATGGAGA3’, R2*R 5′TATGACTGGGAGTAGTCAGGAGAGGAGGAA3’ (465 bp). PCR amplification of *Sox2* in WT and heterozygous mice was performed with the following primers: Sox2ATG407 Frw: 5′ATGTATAACATGATGGAGA 3′, Sox21053 Rvs: 5′ CTGGTCATGGTGTTG 3′ (650 bp). *Sox2* ^INV/+^ mice and embryos that carry inverted COIN cassette, were genotyped with INVdiaR2 (R2), INVdia.R4 (R4) (600 bp) and INVdiaF1: 5′ GTTTTCAGGGTGTTGTTTAG 3′, INVdia(eGFP).F3 (F3) (300 bp) set of primers. Sequences for PCR amplification of *Tg(Sox2-cre)* and *Sox2^βgeo2^* were found in MGI (ID: 103270 and 1915777 respectively). All PCR reactions were performed using 0.2 U/µL Taq Polymerase, standard PCR conditions, and 1 M betaine.

### Imaging Analysis

Conventional bright field and fluorescence microscopy was performed under a Leica MZ16FA stereoscope, while the dissection of the embryos took place either in 1X cold PBS or in DMEM medium supplemented with 2 mM glutamine and 0.5 mM penicillin and streptomycin.

### Western Blotting

E14.5 embryos were lysed by sonication and the resulting pellets were washed with PBS and dissolved in cold buffer A (20 mM Tris-HCl, 420 Mm NaCl, 0.2 Mm EDTA, 0.5 mM DTT, 25% glycerol, 0.5 mM PMSF, 1.5 mM MgCl_2_, 0.5% NP40). Incubation at 4°C for 15 min and centrifugation for 15 min at 10,000×g followed. The supernatant was recovered and the protein concentration was determined by BCA Protein Assay Reagent (bicinchoninic acid) according to the instructions of the manufacturer (Thermo Scientific Pierce BCA Protein Assay Kit). Proteins (50 µg per lane) were separated on 10% SDS-polyacrylamide gel, transferred to nitrocellulose membrane and membrane was blocked in western blot blocking buffer (5% milk, 10 mM Tris-HCl pH 7.6, 0.15 mM NaCl, 0.05% Tween-20) for 2h at RT, incubated o/n with the primary antibody at 4°C. Sox2 rabbit Polyclonal IgG, goat anti-GFP polyclonal antibody (Santa Cruz Biotechnology, Inc, Santa Cruz, CA, USA) and goat β-actin polyclonal antibody (Cell Signaling Technology, Inc, Danvers, MA, USA) were used (1∶1000). After extensive washing in TBST.1 (10 mM Tris-HCl, 0.15 mM NaCl, 0.1% Tween-20), goat anti-rabbit HRP conjugated secondary antibody was applied (1∶10,000) for 2 h at RT. Proteins were visualized by chemiluminescence detection using ECL (Cell Signaling Technology, Inc., Danvers, MA, USA).

### RNA Analysis

RNA was extracted from E14.5 mouse embryos and subjected to Taqman. Real-time PCR analysis typically, *Gapdh* was used as a control house-keeping gene, although analysis was also performed using *Cyclophlin* and *β-actin* with similar results. For miR1897 analysis, miR16 and Sno135 were used as internal controls. All probes are hydrolysis probes with 5' Fam Fluorophore and 3' quencher (BHQ) (Biosearch Technologies). Probes codes and sequences for each gene are: for mSox2, Applied Biosystems, Inc, TaqMan assay ID: Mm00488369_s1, for mSox2ot, Applied Biosystems, Inc, TaqMan assay ID: Mm01291217_m1, for Lac-Z, FRW: TTTCAGCCGCGCTGTACTGGA, RVS: TGTTGCCACTCGCTTTAATGATG, for eGFP: FRW: TCTTCA AGTCCG CCATGCCCG, RVS: CTACCCCGACCACATGAAGC, for miR1897: Applied Biosystems, Inc, TaqMan assay, Probe sequence: 121199.

### Ethics Statement

All animals were handled in strict accordance with good animal practice as defined by the Animals Act 160/03.05.1991 applicable in Greece, revised according to the 86/609/EEC/24.11.1986 EU directive regarding the proper care and use of laboratory animals and in accordance to the Hellenic License for Animal Experimentation at the BSRC” Alexander Fleming” (Prot. No. 767/28.02.07) issued after protocol approval by the Animal Research Committee of the BSRC “Alexander Fleming” (Prot. No. 2762/03.08.05).

## References

[1] Kamachi Y, Iwafuchi M, Okuda Y, Takemoto T, Uchikawa M, et al (2009) Evolution of non-coding regulatory sequences involved in the developmental process: reflection of differential employment of paralogous genes as highlighted by Sox2 and group B1 Sox genes. Proceedings of the Japan Academy Series B, Physical and biological sciences 85: 55–68. 10.2183/pjab.85.55 PMC3524295 19212098

[2] Brosius J, Gould SJ (1992) On “genomenclature”: a comprehensive (and respectful) taxonomy for pseudogenes and other “junk DNA”. Proceedings of the National Academy of Sciences of the United States of America 89: 10706–10710. 1279691 10.1073/pnas.89.22.10706 PMC50410

[3] Gentles AJ, Karlin S (1999) Why are human G-protein-coupled receptors predominantly intronless? Trends in genetics : TIG 15: 47–49. 10098406 10.1016/s0168-9525(98)01648-5

[4] Venter JC, Adams MD, Myers EW, Li PW, Mural RJ, et al (2001) The sequence of the human genome. Science 291: 1304–1351. 11181995 10.1126/science.1058040

[5] Sakharkar KR, Sakharkar MK, Culiat CT, Chow VT, Pervaiz S (2006) Functional and evolutionary analyses on expressed intronless genes in the mouse genome. FEBS letters 580: 1472–1478. 16469316 10.1016/j.febslet.2006.01.070

[6] Sakharkar MK, Perumal BS, Lim YP, Chern LP, Yu Y, et al (2005) Alternatively spliced human genes by exon skipping–a database (ASHESdb). In silico biology 5: 221–225. 15984933

[7] Hill AE, Sorscher EJ (2006) The non-random distribution of intronless human genes across molecular function categories. FEBS letters 580: 4303–4305. 16842783 10.1016/j.febslet.2006.06.051

[8] Friend K, Lovejoy AF, Steitz JA (2007) U2 snRNP binds intronless histone pre-mRNAs to facilitate U7-snRNP-dependent 3' end formation. Molecular cell 28: 240–252. 17964263 10.1016/j.molcel.2007.09.026 PMC2149891

[9] Medlin J, Scurry A, Taylor A, Zhang F, Peterlin BM, et al (2005) P-TEFb is not an essential elongation factor for the intronless human U2 snRNA and histone H2b genes. The EMBO journal 24: 4154–4165. 16308568 10.1038/sj.emboj.7600876 PMC1356315

[10] Maquat LE, Li X (2001) Mammalian heat shock p70 and histone H4 transcripts, which derive from naturally intronless genes, are immune to nonsense-mediated decay. RNA 7: 445–456. 11333024 10.1017/s1355838201002229 PMC1370100

[11] Huang Y, Carmichael GG (1997) The mouse histone H2a gene contains a small element that facilitates cytoplasmic accumulation of intronless gene transcripts and of unspliced HIV-1-related mRNAs. Proceedings of the National Academy of Sciences of the United States of America 94: 10104–10109. 9294170 10.1073/pnas.94.19.10104 PMC23318

[12] Schilham MW, van Eijk M, van de Wetering M, Clevers HC (1993) The murine Sox-4 protein is encoded on a single exon. Nucleic acids research 21: 2009. 8493110 10.1093/nar/21.8.2009 PMC309444

[13] Elkouris M, Balaskas N, Poulou M, Politis PK, Panayiotou E, et al (2011) Sox1 maintains the undifferentiated state of cortical neural progenitor cells via the suppression of Prox1-mediated cell cycle exit and neurogenesis. Stem Cells 29: 89–98. 21280160 10.1002/stem.554

[14] Remboutsika E, Elkouris M, Iulianella A, Andoniadou CL, Poulou M, et al (2011) Flexibility of neural stem cells. Front Physiol 2: 16. 21516249 10.3389/fphys.2011.00016 PMC3079860

[15] Kirby PJ, Waters PD, Delbridge M, Svartman M, Stewart AN, et al (2002) Cloning and mapping of platypus SOX2 and SOX14: insights into SOX group B evolution. Cytogenetic and genome research 98: 96–100. 12584449 10.1159/000068539

[16] Behbahaninia M, Ramey WL, Sindhwani MK, Kalani MY (2011) Differential expression of pluripotency factors Sox2 and Oct4 regulate neuronal and mesenchymal lineages. Neurosurgery 69: N19. 10.1227/01.neu.0000405596.78460.20 21900806

[17] Yoon DS, Kim YH, Jung HS, Paik S, Lee JW (2011) Importance of Sox2 in maintenance of cell proliferation and multipotency of mesenchymal stem cells in low-density culture. Cell proliferation 44: 428–440. 21951286 10.1111/j.1365-2184.2011.00770.x PMC6495637

[18] Parrinello S, Napoli I, Ribeiro S, Digby PW, Fedorova M, et al (2010) EphB signaling directs peripheral nerve regeneration through Sox2-dependent Schwann cell sorting. Cell 143: 145–155. 20869108 10.1016/j.cell.2010.08.039 PMC3826531

[19] Avilion AA, Nicolis SK, Pevny LH, Perez L, Vivian N, et al (2003) Multipotent cell lineages in early mouse development depend on SOX2 function. Genes & development 17: 126–140. 12514105 10.1101/gad.224503 PMC195970

[20] Cavallaro M, Mariani J, Lancini C, Latorre E, Caccia R, et al (2008) Impaired generation of mature neurons by neural stem cells from hypomorphic Sox2 mutants. Development 135: 541–557. 18171687 10.1242/dev.010801

[21] Taranova OV, Magness ST, Fagan BM, Wu Y, Surzenko N, et al (2006) SOX2 is a dose-dependent regulator of retinal neural progenitor competence. Genes & development 20: 1187–1202. 16651659 10.1101/gad.1407906 PMC1472477

[22] Donner AL, Episkopou V, Maas RL (2007) Sox2 and Pou2f1 interact to control lens and olfactory placode development. Developmental biology 303: 784–799. 17140559 10.1016/j.ydbio.2006.10.047 PMC3276313

[23] Okubo T, Pevny LH, Hogan BL (2006) Sox2 is required for development of taste bud sensory cells. Genes & development 20: 2654–2659. 17015430 10.1101/gad.1457106 PMC1578692

[24] Que J, Okubo T, Goldenring JR, Nam KT, Kurotani R, et al (2007) Multiple dose-dependent roles for Sox2 in the patterning and differentiation of anterior foregut endoderm. Development 134: 2521–2531. 17522155 10.1242/dev.003855 PMC3625644

[25] Ferri AL, Cavallaro M, Braida D, Di Cristofano A, Canta A, et al (2004) Sox2 deficiency causes neurodegeneration and impaired neurogenesis in the adult mouse brain. Development 131: 3805–3819. 15240551 10.1242/dev.01204

[26] Dabdoub A, Puligilla C, Jones JM, Fritzsch B, Cheah KS, et al (2008) Sox2 signaling in prosensory domain specification and subsequent hair cell differentiation in the developing cochlea. Proceedings of the National Academy of Sciences of the United States of America 105: 18396–18401. 19011097 10.1073/pnas.0808175105 PMC2587543

[27] Amaral PP, Neyt C, Wilkins SJ, Askarian-Amiri ME, Sunkin SM, et al (2009) Complex architecture and regulated expression of the Sox2ot locus during vertebrate development. RNA 15: 2013–2027. 19767420 10.1261/rna.1705309 PMC2764477

[28] Griffiths-Jones S, Grocock RJ, van Dongen S, Bateman A, Enright AJ (2006) miRBase: microRNA sequences, targets and gene nomenclature. Nucleic acids research 34: D140–144. 16381832 10.1093/nar/gkj112 PMC1347474

[29] Barrand S, Andersen IS, Collas P (2010) Promoter-exon relationship of H3 lysine 9, 27, 36 and 79 methylation on pluripotency-associated genes. Biochem Biophys Res Commun 401: 611–617. 20920475 10.1016/j.bbrc.2010.09.116

[30] Barrand S, Collas P Chromatin states of core pluripotency-associated genes in pluripotent, multipotent and differentiated cells. Biochem Biophys Res Commun 391: 762–767. 19944068 10.1016/j.bbrc.2009.11.134

[31] Alonso MM, Diez-Valle R, Manterola L, Rubio A, Liu D, et al. Genetic and epigenetic modifications of Sox2 contribute to the invasive phenotype of malignant gliomas. PLoS One 6: e26740. 10.1371/journal.pone.0026740 PMC3206066 22069467

[32] Farthing CR, Ficz G, Ng RK, Chan CF, Andrews S, et al (2008) Global mapping of DNA methylation in mouse promoters reveals epigenetic reprogramming of pluripotency genes. PLoS Genet 4: e1000116. 18584034 10.1371/journal.pgen.1000116 PMC2432031

[33] Wiebe MS, Wilder PJ, Kelly D, Rizzino A (2000) Isolation, characterization, and differential expression of the murine Sox-2 promoter. Gene 246: 383–393. 10767561 10.1016/s0378-1119(00)00086-x

[34] Funabashi H, Takatsu M, Saito M, Matsuoka H (2010) Sox2 regulatory region 2 sequence works as a DNA nuclear targeting sequence enhancing the efficiency of an exogenous gene expression in ES cells. Biochem Biophys Res Commun 400: 554–558. 20807503 10.1016/j.bbrc.2010.08.098

[35] Iwafuchi-Doi M, Yoshida Y, Onichtchouk D, Leichsenring M, Driever W, et al (2011) The Pou5f1/Pou3f-dependent but SoxB-independent regulation of conserved enhancer N2 initiates Sox2 expression during epiblast to neural plate stages in vertebrates. Dev Biol 352: 354–366. 21185279 10.1016/j.ydbio.2010.12.027

[36] Miyagi S, Nishimoto M, Saito T, Ninomiya M, Sawamoto K, et al (2006) The Sox2 regulatory region 2 functions as a neural stem cell-specific enhancer in the telencephalon. J Biol Chem 281: 13374–13381. 16547000 10.1074/jbc.M512669200

[37] Miyagi S, Saito T, Mizutani K, Masuyama N, Gotoh Y, et al (2004) The Sox-2 regulatory regions display their activities in two distinct types of multipotent stem cells. Mol Cell Biol 24: 4207–4220. 15121842 10.1128/MCB.24.10.4207-4220.2004 PMC400473

[38] Sikorska M, Sandhu JK, Deb-Rinker P, Jezierski A, Leblanc J, et al (2008) Epigenetic modifications of SOX2 enhancers, SRR1 and SRR2, correlate with in vitro neural differentiation. J Neurosci Res 86: 1680–1693. 18293417 10.1002/jnr.21635

[39] Tomioka M, Nishimoto M, Miyagi S, Katayanagi T, Fukui N, et al (2002) Identification of Sox-2 regulatory region which is under the control of Oct-3/4-Sox-2 complex. Nucleic Acids Res 30: 3202–3213. 12136102 10.1093/nar/gkf435 PMC135755

[40] Griffiths-Jones S (2006) miRBase: the microRNA sequence database. Methods in molecular biology 342: 129–138. 16957372 10.1385/1-59745-123-1:129

[41] Fantes J, Ragge NK, Lynch SA, McGill NI, Collin JR, et al (2003) Mutations in SOX2 cause anophthalmia. Nature genetics 33: 461–463. 12612584 10.1038/ng1120

[42] Economides AN, Frendewey D, Yang P, Dominguez MG, Dore AT, et al.. (2012) Conditionals-by-Inversion: a novel universal method for the generation of conditional alleles. in preparation. 10.1073/pnas.1217812110 PMC3752204 23918385

[43] Albert H, Dale EC, Lee E, Ow DW (1995) Site-specific integration of DNA into wild-type and mutant lox sites placed in the plant genome. Plant J 7: 649–659. 7742860 10.1046/j.1365-313x.1995.7040649.x

[44] Ekonomou A, Kazanis I, Malas S, Wood H, Alifragis P, et al (2005) Neuronal migration and ventral subtype identity in the telencephalon depend on SOX1. PLoS biology 3: e186. 15882093 10.1371/journal.pbio.0030186 PMC1110909

[45] Nagy A (2000) Cre recombinase: the universal reagent for genome tailoring. Genesis 26: 99–109. 10686599

[46] Ringrose L, Chabanis S, Angrand PO, Woodroofe C, Stewart AF (1999) Quantitative comparison of DNA looping in vitro and in vivo: chromatin increases effective DNA flexibility at short distances. Embo J 18: 6630–6641. 10581237 10.1093/emboj/18.23.6630 PMC1171726

[47] Testa G, Schaft J, van der Hoeven F, Glaser S, Anastassiadis K, et al (2004) A reliable lacZ expression reporter cassette for multipurpose, knockout-first alleles. Genesis 38: 151–158. 15048813 10.1002/gene.20012

[48] Skarnes WC, Rosen B, West AP, Koutsourakis M, Bushell W, et al (2011) A conditional knockout resource for the genome-wide study of mouse gene function. Nature 474: 337–342. 21677750 10.1038/nature10163 PMC3572410

[49] Seoighe C, Korir PK (2011) Evidence for intron length conservation in a set of mammalian genes associated with embryonic development. BMC Bioinformatics 12 Suppl 9 S16. 10.1186/1471-2105-12-S9-S16 PMC3283306 22151910

[50] Swinburne IA, Miguez DG, Landgraf D, Silver PA (2008) Intron length increases oscillatory periods of gene expression in animal cells. Genes Dev 22: 2342–2346. 18703678 10.1101/gad.1696108 PMC2532923

[51] Swinburne IA, Silver PA (2008) Intron delays and transcriptional timing during development. Dev Cell 14: 324–330. 18331713 10.1016/j.devcel.2008.02.002 PMC2825037

[52] Miyagi S, Masui S, Niwa H, Saito T, Shimazaki T, et al (2008) Consequence of the loss of Sox2 in the developing brain of the mouse. FEBS letters 582: 2811–2815. 18638478 10.1016/j.febslet.2008.07.011

[53] Smith AN, Miller LA, Radice G, Ashery-Padan R, Lang RA (2009) Stage-dependent modes of Pax6-Sox2 epistasis regulate lens development and eye morphogenesis. Development 136: 2977–2985. 19666824 10.1242/dev.037341 PMC2723069

[54] Favaro R, Valotta M, Ferri AL, Latorre E, Mariani J, et al (2009) Hippocampal development and neural stem cell maintenance require Sox2-dependent regulation of Shh. Nature neuroscience 12: 1248–1256. 19734891 10.1038/nn.2397

[55] Valenzuela DM, Murphy AJ, Frendewey D, Gale NW, Economides AN, et al (2003) High-throughput engineering of the mouse genome coupled with high-resolution expression analysis. Nature biotechnology 21: 652–659. 10.1038/nbt822 12730667

[56] Zhang Y, Buchholz F, Muyrers JP, Stewart AF (1998) A new logic for DNA engineering using recombination in Escherichia coli. Nat Genet 20: 123–128. 9771703 10.1038/2417

